# Outcome of cardiac surgery in patients with low preoperative ejection fraction

**DOI:** 10.1186/s12871-016-0271-5

**Published:** 2016-10-18

**Authors:** Marina Pieri, Alessandro Belletti, Fabrizio Monaco, Antonio Pisano, Mario Musu, Veronica Dalessandro, Giacomo Monti, Gabriele Finco, Alberto Zangrillo, Giovanni Landoni

**Affiliations:** 1 Department of Anesthesia and Intensive Care, IRCCS San Raffaele Scientific Institute, Via Olgettina 60, 20132 Milan, Italy; 2 Cardiac Anesthesia and Intensive Care Unit, Monaldi Hospital A.O.R.N. “Dei Colli”, Naples, Italy; 3 Department of Medical Sciences “M. Aresu”, University of Cagliari, Cagliari, Italy; 4 Vita-Salute San Raffaele University, Milan, Italy

**Keywords:** Cardiac surgery, Left ventricular dysfunction, Low cardiac output syndrome, Mitral valve surgery, Left ventricular ejection fraction, Coronary artery bypass graft, Intensive care, Anesthesia, Mortality

## Abstract

**Background:**

In patients undergoing cardiac surgery, a reduced preoperative left ventricular ejection fraction (LVEF) is common and is associated with a worse outcome. Available outcome data for these patients address specific surgical procedures, mainly coronary artery bypass graft (CABG). Aim of our study was to investigate perioperative outcome of surgery on patients with low pre-operative LVEF undergoing a broad range of cardiac surgical procedures.

**Methods:**

Data from patients with pre-operative LVEF ≤40 % undergoing cardiac surgery at a university hospital were reviewed and analyzed. A subgroup analysis on patients with pre-operative LVEF ≤30 % was also performed.

**Results:**

A total of 7313 patients underwent cardiac surgery during the study period. Out of these, 781 patients (11 %) had a pre-operative LVEF ≤40 % and were included in the analysis. Mean pre-operative LVEF was 33.9 ± 6.1 % and in 290 patients (37 %) LVEF was ≤30 %. The most frequently performed operation was CABG (31 % of procedures), followed by mitral valve surgery (22 %) and aortic valve surgery (19 %). Overall perioperative mortality was 5.6 %. Mitral valve surgery was more frequent among patients who did not survive, while survivors underwent more frequently CABG. Post-operative myocardial infarction occurred in 19 (2.4 %) of patients, low cardiac output syndrome in 271 (35 %). Acute kidney injury occurred in 195 (25 %) of patients. Duration of mechanical ventilation was 18 (12–48) hours. Incidence of complications was higher in patients with LVEF ≤30 %. Stepwise multivariate analysis identified chronic obstructive pulmonary disease, pre-operative insertion of intra-aortic balloon pump, and pre-operative need for inotropes as independent predictors of mortality among patients with LVEF ≤40 %.

**Conclusions:**

We confirmed that patients with low pre-operative LVEF undergoing cardiac surgery are at higher risk of post-operative complications. Cardiac surgery can be performed with acceptable mortality rates; however, mitral valve surgery, was found to be associated with higher mortality rates in this population. Accurate selection of patients, risk/benefit evaluation, and planning of surgical and anesthesiological management are mandatory to improve outcome.

**Electronic supplementary material:**

The online version of this article (doi:10.1186/s12871-016-0271-5) contains supplementary material, which is available to authorized users.

## Background

Low preoperative left ventricular ejection fraction (LVEF) is common in patients undergoing cardiac surgery, especially those scheduled for coronary artery bypass graft (CABG) surgery. Despite improvements in medical therapy and surgical techniques, management of patients with moderate or severe left ventricular dysfunction undergoing cardiac surgery remains challenging [1, 2]. As known, patients with low LVEF are at a higher risk for postoperative complications and mortality after cardiac surgery [1]. Therefore, an early recognition of patients at risk for a worse outcome plays a pivotal role in the decision making process, allowing the prompt institution of an adequate support [3]. Several perioperative variables have been purposed as predictors of mortality [4–8], including acute renal failure [9] and pneumonia [10], and are currently applied in everyday clinical practice [10] to identify patients at higher risk. Low EF is per se the strongest predictor of a poor outcome and is included in all scoring system currently available. Indeed, low LVEF is associated to postoperative low cardiac output syndrome (LCOS), need for inotropic support [11, 12], acute renal failure [9, 13, 14], respiratory failure [1], pneumonia [10], atrial fibrillation [15], stroke, sepsis or endocarditis, deep sternal wound infection, bleeding requiring reoperation and gastrointestinal bleeding [1]. However, the outcome after cardiac surgery has improved over time leading to a significant decrease of the performance of the currently available scores. There is a currently unmet need for more sophisticated preoperative predictive parameters, which may help to further stratify patients with impaired cardiac function, that are nevertheless candidate to undergo cardiac surgery. Indeed several biological and procedural variables, the constant evolution in both practice of surgery and perioperative medicine, the volume of activity of the hospital should somehow be taken into consideration [16], together with EF.

Aim of the present study was therefore to assess the mortality rate in high-risk patients with low EF (<40 %) undergoing cardiac surgery and to identify the risk factors associated with a worse outcome in a national referral cardiac surgery center with a high volume of surgical activity.

## Methods

The study was performed in accordance with the Declaration of Helsinki and its amendments. After approval by the local Ethical Committee (OSR Ethical Committee, 38 AN CCH 31-10-2013) data from all patients with LVEF ≤ 40 % who underwent cardiac surgery at San Raffaele Scientific Institute over a 6 years’ period were collected. No specific written consent was obtained for this retrospective observational study since all patients’ data were anonymized and de-identified prior to analysis. However, all patients signed a written consent for the use of their data for scientific purposes.

All patients underwent transthoracic and/or transesophagel echocardiography as part of routine pre-operative assessment. The examination was performed by a cardiologist trained in perioperative echocardiography, usually the day before surgery. The threshold for data analysis (40 %) followed the 2015 recommendation from the American Society of Echocardiography and the European Association of Cardiovascular Imaging [17], which further identified an LVEF value of 30 % as the cut off of severely depressed cardiac function.

All patients underwent cardiac surgery under general anesthesia and at the end of the surgery were transferred to the intensive care unit (ICU). A standard premedication with morphine 0.1 mg/kg subcutaneously and scopolamine 0.25 mg intramuscularly one hour before surgery was administered. General anesthesia was induced with an intravenous bolus of propofol, fentanyl and muscle relaxant and maintained with fentanyl, muscle relaxants and either halogenates or propofol (or both). An intravenous infusion of tranexamic acid was administered intraoperatively: 1 g in 20 min followed by a 400 mg/h infusion. A temperature of 32–34 °C was maintained during cardiopulmonary bypass (CPB) and myocardial protection during aortic cross clamping was performed by anterograde and/or retrograde cold cardioplegia. Unfractionated heparin (at a starting dose of 3 mg/kg) was administered in order to maintain an activated clotting time (ACT) of more than 480 s during CPB. Heparin was reversed with protamine in a 1:1 ratio. The target mean arterial pressure during CPB was 65 mmHg.

After surgery, patients were transferred to the ICU under sedation with propofol. Weaning from mechanical ventilation was started, in the absence of hemodynamic instability and major bleeding, as soon as normothermia and an adequate level of consciousness were achieved. Antibiotic prophylaxis was performed with intravenous cephazolin. Standard therapy also included hydration, antiacids and diuretics, as well as inotropic drugs and mechanical circulatory support devices when required by the hemodynamic conditions.

Myocardial infarction was defined according to the Consensus Conference for the Universal Definition of Myocardial Infarction [18]. Low cardiac output syndrome was defined as arterial hypotension (systolic blood pressure <100 mmHg) with signs of organ hypoperfusion (decreased urine output, lactic acidosis,..) and cardiac index below 2 l/min/m^2^ despite adequate fluid replacement. Cardiogenic shock was defined according to the IABP-SHOCK Trial [19]. Acute kidney injury (AKI) was diagnosed in the presence of an increase in serum creatinine of more than 50 % or a decrease in the glomerular filtration rate (GFR) of more than 25 % as compared to preoperative values [20]. The Cockroft-Gault equation was used to estimate GFR [21]. Age-Creatinine-Ejection Fraction (ACEF) score was calculated [22]. We defined “redo” a patient who had already undergone sternotomy for cardiac surgery. Postoperative atrial fibrillation (AF) was defined as a new onset of AF requiring pharmacological or electrical cardioversion during the ICU stay.

### Statistical analysis

Data were stored electronically and analyzed using the 9.2 version of the SAS software (SAS Institute Inc., Cary, NC, USA). Categorical variables are reported as numbers (percent), whereas continuous variables are expressed as mean ± standard deviation (SD) or as median (interquartile range) according to the Kolmogorov-Smirnov test. The Fisher’s test was used to calculate *p* values between two groups for categorical variables. Multiple logistic regression was used to identify independent predictors of mortality. A stepwise selection method was used for death (dichotomous variable), with COPD, Pre-operative intraaortic balloon pump (IABP), Pre-operative inotropes for patients with FE ≤ 40 % and with Pre-operative renal failure and Mitral valve surgery for patients with FE ≤ 30 %. The area under the ROC curves of the two predictive models was also calculated.

## Results

Out of 7357 patients undergoing cardiac surgery in the study period, 7313 had data on preoperative LVEF. Of these, 781 patients (11 %) had preoperative LVEF ≤ 40 % and were included in the study. Baseline characteristics, comorbidities, type of operation and intraoperative management of the study population are reported in Table 1.

**Table 1:** Baseline and intra-operative characteristics of patients with ejection fraction ≤40 % who underwent cardiac surgery: comparisons between survivors and dead patients

Variable	Total (*N* = 781)	Survivors (*N* = 737)	Dead (*N* = 44)	*P*-value
Preoperative characteristics				
Gender (Male), *n*	597 (76 %)	569 (77 %)	28 (64 %)	0.04
Age, years	65.4 ± 10.3	65.3 ± 10.4	68.4 ± 9.4	0.0575
Height, cm	169.6 ± 8.1	169.7 ± 8.0	167.6 ± 9.4	0.2
Weight, kg	73.7 ± 13.4	73.8 ± 13.3	71.2 ± 14.6	0.2
BMI	25.6 ± 4.0	25.6 ± 4.0	25.4 ± 4.4	0.8
Comorbidity				
> COPD, *n*	235 (30 %)	210 (28 %)	25 (57 %)	<0.001
> Preoperative EF, %	33.9 ± 6.1	34.0 ± 6.1	31.8 ± 6.0	0.01
> Preoperative EF ≤ 40 %, *n*	781 (100 %)			
> Preoperative EF ≤ 30 %, *n*	290 (37 %)			
> Peripheral vasculopathy, *n*	196 (25 %)	183 (25 %)	13 (30 %)	0.5
> Arterial hypertension, *n*	423 (54 %)	402 (55 %)	21 (48 %)	0.4
> Type II diabetes mellitus, *n*	159 (20 %)	151 (20 %)	8 (18 %)	0.7
> Carotid stenosis, *n*	71 (9.1 %)	68 (9.2 %)	3 (6.8 %)	0.8
> Angina, *n*	112 (14 %)	104 (14 %)	8 (18 %)	0.5
> Previous AMI, *n*	243 (31 %)	227 (31 %)	16 (36 %)	0.4
> Previous TIA or stroke, *n*	61 (7.8 %)	58 (7.9 %)	3 (6.8 %)	0.99
> Previous vascular surgery, *n*	37 (4.7 %)	37 (5 %)	0 (0 %)	0.3
> Standard EuroSCORE	6 (4–8)	6 (4–8)	8 (6–10)	<0.001
> ACEF score	2.08 (1.77–2.47)	2.06 (1.75–2.44)	2.43 (2.08–3.16)	<0.001
> ACEF risk	5.59 (3.89–8.78)	5.46 (3.81–8.56)	8.45 (5.61–18.52)	<0.001
> Endocarditis, *n*	22 (2.8 %)	20 (2.7 %)	2 (4.5)	0.4
> Creatinine clearance, ml/h	65.1 (49.2–82.6)	65.67 (50.23–83.33)	49.1 (39.66–69.27)	0.002
> Chronic renal failure, *n*	149 (19 %)	134 (18 %)	15 (34 %)	0.009
> Dialysis, *n*	13 (1.7 %)	12 (1.6 %)	1 (2.3 %)	0.5
NYHA				0.003
> I	51 (6.5 %)	51 (6.9 %)	0 (0 %)	
> II	208 (27 %)	201 (27 %)	7 (16 %)	
> III	276 (35 %)	267 (36 %)	9 (20 %)	
> IV	43 (5.5 %)	36 (4.9 %)	7 (16 %)	
Timing of surgery				0.2
> Emergency, *n*	18 (2.3 %)	16 (2.2 %)	2 (4.5 %)	
> Urgency, *n*	129 (17 %)	119 (16 %)	10 (23 %)	
> Election, *n*	634 (81 %)	602 (82 %)	32 (73 %)	
Redo surgery, *n*	81 (10 %)	73 (9.9 %)	8 (18 %)	0.08
Preoperative IABP, *n*	135 (17 %)	121 (16 %)	14 (32 %)	0.009
Preoperative inotropes, *n*	17 (2.2 %)	11 (1.5 %)	6 (14 %)	<0.001
Chronic therapy				
> Antiplatelets, *n*	267 (34 %)	258 (35 %)	9 (20 %)	0.048
> Diuretics, *n*	503 (64 %)	470 (64 %)	33 (75 %)	0.13
> Beta-blockers, *n*	365 (47 %)	353 (48 %)	12 (27 %)	0.008
> Antibiotics, *n*	38 (4.9 %)	34 (4.6 %)	4 (9.1 %)	0.16
> Calcium channel blockers, *n*	128 (16 %)	121 (16 %)	7 (16 %)	0.9
> Nitrates, *n*	217 (28 %)	205 (28 %)	12 (27 %)	0.9
> ACE inhibitors, *n*	496 (64 %)	473 (64 %)	23 (52 %)	0.11
> Oral anticoagulants, *n*	135 (17 %)	130 (18 %)	5 (11 %)	0.4
> Heparin, *n*	61 (7.8 %)	57 (7.7 %)	4 (9.1 %)	0.8
Creatinine, mg/dl	1.2 ± 0.9	1.2 ± 0.9	1.5 ± 1.1	0.004
Bilirubin, mg/dl	0.8 (0.57–1.1)	0.8 (0.57–1.04)	0.94 (0.63–1.4)	0.1
Surgical interventions				
CABG, *n*	390 (31 %)	373 (31 %)	17 (23 %)	0.12
> Isolated CABG, *n*	189 (15 %)	185 (16 %)	4 (5.4 %)	0.02
Mitral valve surgery, *n*	282 (22 %)	258 (22 %)	24 (32 %)	0.009
> Isolated mitral valve surgery, *n*	90 (7.1 %)	84 (7.1 %)	6 (8.1 %)	0.7
> Mitral valve replacement, *n*	126 (10 %)	117 (9.9 %)	9 (12 %)	0.4
> Mitral valve repair, *n*	156 (12 %)	141 (12 %)	15 (20 %)	0.02
Aortic valve surgery, *n*	241 (19 %)	227 (19 %)	14 (19 %)	0.9
> Isolated aortic valve surgery, *n*	81 (6.4 %)	76 (6.4 %)	5 (6.8 %)	0.8
> Aortic valve replacement, *n*	241 (19 %)	227 (19 %)	14 (19 %)	0.9
> Aortic valve repair, *n*	1 (0.08 %)	1 (0.08 %)	0 (0 %)	0.99
Tricuspid valve surgery, *n*	96 (7.6 %)	91 (7.7 %)	5 (6.8 %)	0.99
> Isolated tricuspid valve surgery, *n*	3 (0.24 %)	3 (0.25 %)	0 (0 %)	0.99
> Tricuspid valve replacement, *n*	6 (0.48 %)	6 (0.51 %)	0 (0 %)	0.99
> Tricuspid valve repair, *n*	90 (7.1 %)	85 (7.2 %)	5 (6.8 %)	0.99
Pulmonic valve surgery, *n*	1 (0.08 %)	1 (0.08 %)	0 (0 %)	0.99
> Isolated pulmonic valve surgery, *n*	1 (0.08 %)	1 (0.08 %)	0 (0 %)	0.99
Surgery on ascending aorta, *n*	83 (6.6 %)	76 (6.4 %)	7 (9.5 %)	0.2
> Isolated surgery on ascending aorta, *n*	6 (0.48 %)	6 (0.51 %)	0 (0 %)	0.99
Left ventricle surgery, *n*	77 (6.1 %)	76 (6.4 %)	1 (1.4 %)	0.11
> Isolated left ventricle surgery, *n*	12 (0.95 %)	12 (1 %)	0 (0 %)	0.99
Intraoperative management				
CPB, *n*	696 (91 %)	656 (89 %)	40 (91 %)	0.4
Duration of aortic cross clamping, min	61 (48–78)	61 (47–78)	69.5 (51–78)	0.3
Duration of CPB, min	85 (65–102)	84 (65–101)	95 (70–114)	0.3

*ACEF* age-creatinine-ejection fraction, *AMI* acute myocardial infarction, *BMI* body mass index, *CABG* coronary artery bypass graft, *COPD* chronic obstructive pulmonary disease, *CPB* cardiopulmonary bypass, *EF* ejection fraction, *IABP* intra-aortic balloon pump, *NYHA* New York Heart Association, *TIA* transient ischemic attack

Mean age was 65.4 ± 10.3 years, and 76 % of patients were male. Mean preoperative LVEF was 33.9 ± 6.1 %.

The most common intervention performed was CABG, followed by mitral valve surgery (either replacement or repair), and aortic valve replacement. Three-hundred sixty six patients (47 %) underwent combined surgical procedures.

**Fig. 1:**
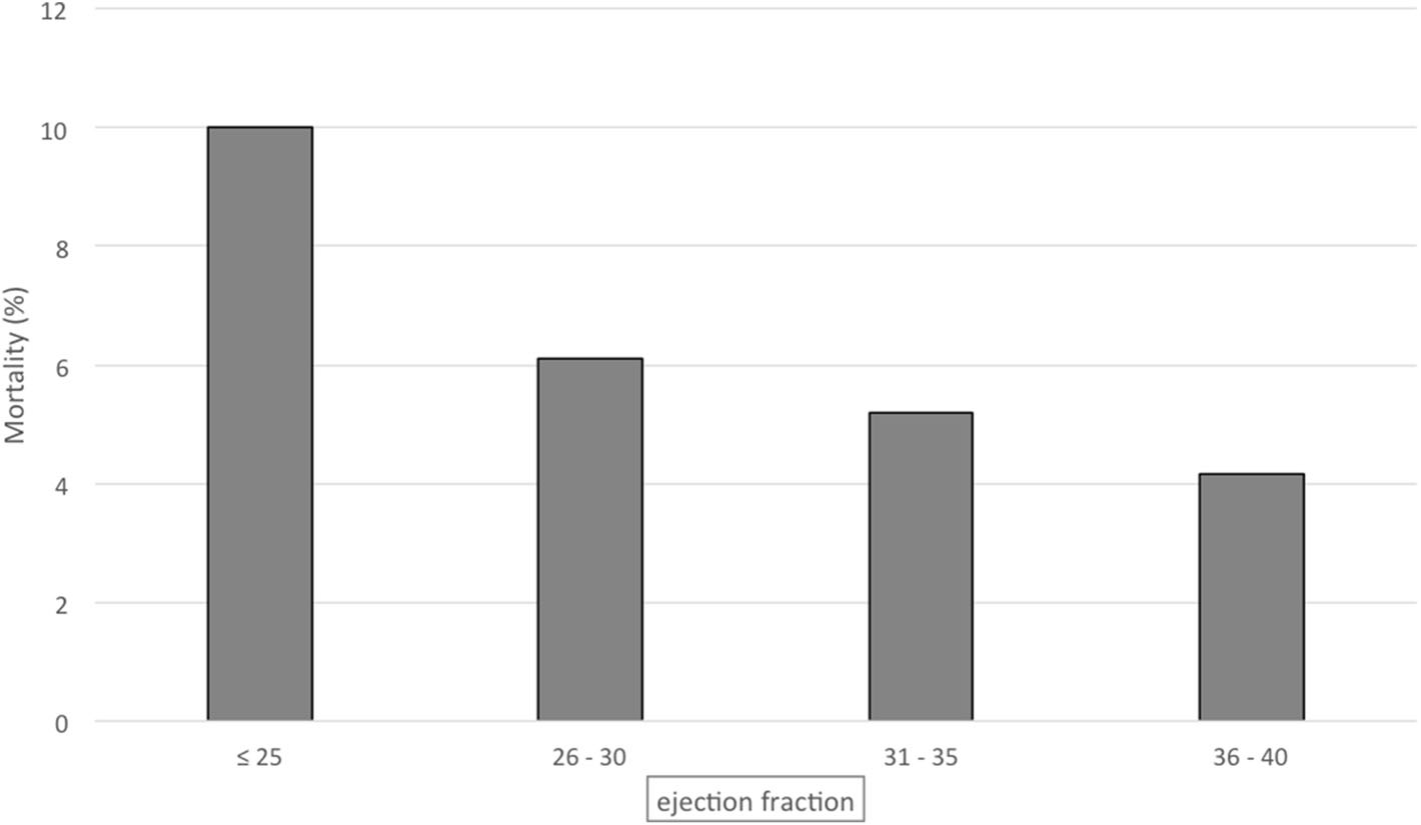
Mortality in the different classes of ejection fraction

Postoperative outcomes are reported in Table 2. Among patients with LVEF ≤ 40 %, mortality was 5.6 % and was consistent with preoperative predictions (mean EuroSCORE was six and mean ACEF score was 5.59). Mortality rates for the different LVEF classes are presented in Fig. 1. As expected, mortality risk increases as LVEF decreases. In the study cohort, mitral valve surgery was the most common operation performed among non-survivors compared with survivors (32 vs. 22 %, *p* = 0.009). Conversely, isolated CABG was the most common operation performed among survivors (16 vs. 5.4 %, *p* = 0.02). Survivors had significantly shorter ICU length of stay (LOS) (3 vs. 12 days, *p* < 0.001), hospital LOS (7 vs. 15.5 days, *p* < 0.001), duration of mechanical ventilation (18 vs. 88 h, *p* < 0.001), lower need for renal replacement therapy (RRT) (3.3 % vs. 50 %, *p* < 0.001), lower troponin peak (7.83 vs. 21.32, *p* < 0.001), and less need for blood transfusions (31 vs. 70 %, *p* < 0.001). Regarding postoperative complications, patients who died had a significantly higher rate of LCOS (75 vs. 32 %, *p* < 0.001), cardiogenic shock (55 vs. 3.4 %, *p* < 0.001), AKI (86 vs. 21 %, *p* < 0.001), sepsis (11 vs. 2.7 %, *p* = 0.01), and severe pulmonary dysfunction (27 vs. 2.7 %, *p* < 0.001).

**Table 2:** Post-operative complications and outcome data of patients with ejection ≤40 % who underwent cardiac surgery: comparisons between survived and dead patients

Variable	Total (*N* = 781)	Survived (*N* = 737)	Dead (*N* = 44)	*P*-value
Post-operative complications				
Post-operative AMI, *n*	19 (2.4 %)	17 (2.3 %)	2 (4.5 %)	0.3
Post-operative peak troponin value, ng/ml	8.03 (4.4–15)	7.83 (4.3–14.35)	21.32 (8.07–29.73)	<0.001
Post-operative AF, *n*	202 (26 %)	191 (26 %)	11 (25 %)	0.9
LCOS, n	271 (35 %)	238 (32 %)	33 (75 %)	<0.001
Inotropes more than 48 h, *n*	233 (30 %)	204 (28 %)	29 (66 %)	<0.001
Cardiogenic shock, *n*	49 (6.3 %)	25 (3.4 %)	24 (55 %)	<0.001
Post-operative peak creatinine value, mg/dl	1.3 ± 0.9	1.3 ± 0.9	1.5 ± 1.1	0.004
AKI, *n*	195 (25 %)	157 (21 %)	38 (86 %)	<0.001
RRT, *n*	46 (5.9 %)	24 (3.3 %)	22 (50 %)	<0.001
Bleeding in the first 12 postoperative hours, ml	280 (200–400)	280 (200–400)	260 (180–425)	0.9
Total post-operative bleeding, ml	460 (300–720)	460 (300–720)	585 (455–735)	0.2
Need for blood products transfusion, *n*	263 (34 %)	232 (31 %)	31 (70 %)	<0.001
RBC transfusions, n of units per patient	0 (0–1)	0 (0–1)	3 (0–6.5)	<0.001
FFP transfusions, n of units per patient	0 (0–0)	0 (0–0)	0 (0–3)	<0.001
PLT transfusions, n of units per patient	0 (0–0)	0 (0–0)	0 (0–0.5)	<0.001
Neurological damage type 1, *n*	16 (2 %)	14 (1.9 %)	2 (4.5 %)	0.2
Neurological damage type 2, *n*	22 (2.8 %)	19 (2.6 %)	3 (6.8 %)	0.12
Severe pulmonary dysfunction, *n*	32 (4.1 %)	20 (2.7 %)	12 (27 %)	<0.001
Tracheostomy, *n*	27 (3.5 %)	12 (1.6 %)	15 (34 %)	<0.001
Need for re-intubation, *n*	23 (2.9 %)	13 (1.8 %)	10 (23 %)	< 0.001
Sepsis, *n*	25 (3.2 %)	20 (2.7 %)	5 (11 %)	0.01
Mediastinitis, *n*	6 (0.77 %)	3 (0.41 %)	3 (6.8 %)	0.003
Outcome data				
Duration of MV, hours	18 (12–48)	18 (12–43)	88 (34–288)	<0.001
ICU stay, days	3 (1–5)	3 (1–5)	12 (4–19)	<0.001
Hospital stay, days	7 (5–11)	7 (5–11)	15.5 (8–24)	<0.001
Death, *n*	44 (5.6 %)			

*AF* atrial fibrillation, *AKI* acute kidney injury, *AMI* acute myocardial infarction, *FFP* fresh frozen plasma, *ICU* intensive care unit, *LCOS* low cardiac output syndrome, *MV* mechanical ventilation, *PLT* platelets, *RBC* red blood cells, *RRT* renal replacement therapy

Multivariate analysis identified the following independent predictors of mortality in patients with LVEF ≤ 40 %: history of chronic obstructive pulmonary disease (COPD) (odds ratio [OR] 3.419, 95 % confidence interval [CI] 1.266–9.238, *p* = 0.015), preoperative use of IABP (OR 3.335, 95 % CI 1.258–8.839, *p* = 0.015), and need for inotropes prior to surgery (OR 13.595, 95 % CI 2.852–64.808, *p* = 0.001) (Table 3). The area under the ROC curve was 0.61.

**Table 3:** Independent predictors of mortality (stepwise multivariate analysis)

Variable	Odds ratio	95 % Confidence interval		*P*-value
LVEF ≤40 %				
COPD	3.419	1.266	9.238	0.0153
Pre-operative IABP	3.335	1.258	8.839	0.0154
Pre-operative inotropes	13.595	2.852	64.808	0.0011
LVEF ≤30 %				
Pre-operative renal failure	6.845	1.841	25.450	0.0041
Mitral valve surgery	5.244	1.290	21.322	0.0206

*COPD* chronic obstructive pulmonary disease, *IABP* intra-aortic balloon pump

Two hundred and ninety patients (37.13 %) had a pre-operative LVEF below 30 % (mean, 26.9 ± 3.8 %). Baseline and outcomes data of these patients are shown as supplementary material (Additional file 1: Tables S1 and S2 respectively). Mean age (65.4 ± 9.9 years) was similar to the entire cohort with a prevalence of male.

Although the mortality rate in patients with LVEF ≤ 30 % was higher (7.6 %) than the overall study population it was lower than expected (mean EuroSCORE = 7; mean ACEF mortality risk = 10.19). One hundred-sixty patients (48 %) underwent combined surgery.

The most common operation performed in patients with LVEF ≤ 30 %, who did not-survive was mitral valve surgery (37 % of non-survivors vs. 21 % of survivors, *p* = 0.005). On the contrary, all the 59 patients (12 %) with LVEF <30 % who underwent isolated CABG survived. Unlike the whole cohort of low LVEF patients, in those with LVEF ≤ 30 % there was no statistically significant difference between survivors and non-survivors for the rate of wound infections, mediastinitis, sepsis, serum creatinine peak, hospital LOS, and length of mechanical ventilation. Moreover, no gastrointestinal complications occurred in this subgroup.

The rate of perioperative myocardial infarction was not statistically different between survivors and non-survivors, both in the entire study population and in the subgroup of patients with LVEF ≤ 30 %.

The only two predictors independently associated to mortality in the sub-group of patients with an EF <30 % were preoperative renal failure (defined as pre-operative eGFR < 90 mL/min) and mitral valve surgery (OR 6.845, 95 % CI 1.841–25.45, *p* = 0.004 and OR 5.244, 95 % CI 1.290–21.322, *p* = 0.021, respectively) (Table 3). The area under the ROC curve was 0.53.

As mitral valve surgery was found to be associated with mortality, we performed a further comparison between patients who had undergone isolated mitral valve versus isolated CABG surgery. The mortality rate of patients with EF ≤ 40 % (Table 1) was not different between these two groups (*p* = 0.081), but reached statistical significance in patients with EF ≤30 % (Additional file 1: Table S1).

Baseline descriptive data of patients undergoing mitral valve surgery are reported in Additional file 1: Table S3.

## Discussion

Data from this large cohort of cardiac surgical patients confirmed that low LVEF still represent a common issue in this setting, affecting more than 10 % of patients. This population represents a group at higher surgical risk due to the greatly reduced cardiovascular reserve; for this reason, a comprehensive and insightful preoperative risk stratification, beyond the LVEF value itself, is strongly recommended. In this sense, our experience may add new clues to be implemented into clinical practice.

In the overall cardiac surgical population, CABG is the most frequently performed operation [5, 23], and ischemic cardiomyopathy is the most frequent cause of heart failure associated to a reduced EF [24]. Therefore, most cardiac surgery studies have been led on patients with low LVEF undergoing CABG. On the contrary, very few studies have investigated the outcome of patients with low EF undergoing valve surgery [25–27]. As observed by Hamad et al, the prevalence of low EF is as high as 20 % in patients undergoing CABG [2], while the percentage decrease to 10–15 % in patients undergoing valve surgery [25]. To best of our knowledge this is the first study in which a large heterogeneous population with an EF below 40 % undergoing predominantly valve cardiac surgery in a high volume hospital has been investigated. The percentage of patients with pre-operative EF below 40 % was 11 %, with one-third of patients undergoing CABG, and more than 40 % who underwent valve surgery. The vast majority of studies in patients with low EF undergoing CABG has shown a higher perioperative risk and a better survival after myocardial revascularization. In this setting the long-term benefits clearly overcome an increased peri-operative mortality [28].

On the contrary, there are no conclusive data on the perioperative risk of patients with low EF undergoing valve surgery. The assessment of the perioperative risk in this kind of patients is of particular relevance for both the surgical planning and the intra- and postoperative management. Furthermore, the surgical correction of different valve defects has different impact on early postoperative ventricular function and outcome [29, 30].

The main result of the present study is that, even in patients with valve disease and impaired EF, cardiac surgery can be performed with acceptable mortality rates Namely, the mortality rate in patients with LVEF ≤ 40 % was 5.6 %, while in the subgroup of patients with LVEF ≤ 30 % was 7.6 %. These findings are slightly better than those reported which range between 3 and 10 % in CABG [1, 2, 31] and valve surgery [25–27]. In our high surgery volume tertiary care center the mortality rate is better in patients with the highest degree of ventricular dysfunction (LVEF ≤ 30 %) compared to previous studies, which report a mortality rate as high as 7–10 % [2, 27]. Our results confirm that, in patients with a reduced LVEF (especially in those with LVEF ≤ 30 %), mitral valve surgery is associated with greater risk of an adverse outcome compared to isolated CABG. The prognosis might even be poorer when CABG is associated to mitral valve surgery [5, 32]. In fact, the mitral valve repair and replacement are associated to the highest risk of early postoperative LCOS due to the afterload mismatch, the highest troponin I release [33] and the highest preoperative risk of death [34–37]. Notably, LVEF in the context of moderate-severe and severe mitral regurgitation cannot be relied upon to describe LV systolic function, since part of the left ventricular stroke volume is directed backward (towards the left atrium) and does not contribute to systemic perfusion [38]. Furthermore, the reduction of EF in these patients might often be caused by ischemic cardiomyopathy. In a recent sub-analysis of the Surgical Treatment for Ischemic Heart Failure (STICH) trial, the presence of severe mitral regurgitation was found to be a predictor of 30-day mortality in patients with left ventricular dysfunction undergoing CABG. Interestingly, concomitant mitral valve surgery was associated with better 30-day survival as compared with no mitral valve surgery [31]. Although results from the STICH trial have been challenged by a recent multicenter RCT by Smith and colleagues [39], it should be highlighted that these latter study did not focus on patients with reduced LVEF and mortality was not the primary endpoint in the RCT [39]. Treatment strategies for secondary mitral regurgitation are still a matter of debate, with available studies giving conflicting results [40] and current guidelines providing only weak recommendations [41, 42]. Therefore, optimal management of these patients requires both accurate evaluation and risk stratification and a thorough discussion with the patient of the possible risks and benefits.

In patients with EF < 40 %, mitral valve surgery, preoperative IABP use and need for inotropes, COPD and chronic kidney disease were identified as independent predictors of mortality. The use of pre-operative IABP and inotropes are markers of severity illness rather than primary cause of a worse outcome. Zangrillo et al. have recently shown indeed that IABP improves the outcomes in high risk patients undergoing CABG [43].

It is not surprising that the need of inotropes prior to surgery is the strongest predictors of mortality. Chronic kidney disease and COPD are well known risk factors for both short- and long-term morbidity and mortality after cardiac surgery [44–47]. Our findings clearly show how these conditions acquire an even more considerable relevance in patients who also have a reduced LV function. In the present study the rate of postoperative complications is higher than previously reported in literature. In particular compared with data from recent large studies led in a general cardiac surgical population [48, 49], in this high risk population with low EF we found a higher rate of postoperative AKI, AF and LCOS [36, 37]. On the contrary, we observed a lower rate of myocardial infarction. A possible explanation is that postoperative myocardial infarction after cardiac surgery may be related to inaccurate myocardial protection perioperative hemodynamic instability, a post-operative pro-thrombotic state complexity of coronary revascularization, surgical technical skills rather than LVEF itself [50].

The following limitations have to be considered: first the retrospective design of the study [51]; second it covers a relatively long period during which both the indications to surgery and the perioperative care may have changed; third the lack of a long-term follow-up. The study is monocentric and a validation group was not available. The performance of the two models is not optimal and they might be further improved in larger multicentric studies; however, the cut-offs for analyses were based on relevant clinical parameters and were chosen according to international guidelines [17]. We are aware that LVEF remains among the strongest predictors of clinical outcome after cardiac surgery, and we cannot exclude that other parameters, that were not taken into consideration in the present study, might play a relevant role. Finally, in some cases, isolated CABG surgery was performed, although mitral valve disease was also present: as we cannot provide the rate of occurrence of this phenomenon, we cannot exclude that it might have influenced our results.

## Conclusions

Moderate-to-severe left ventricular dysfunction is a common finding in the general cardiac surgical population. Patients with reduced LVEF undergoing cardiac surgery have a high-risk of postoperative complications, and higher mortality rates, however the surgery can be performed with relatively low mortality rate. Among the different cardiac surgical procedures patients with low EF undergoing mitral valve surgery show the highest mortality. Accurate preoperative evaluation and risk stratification are of paramount importance in these patients, and careful perioperative management is mandatory.

## Additional file

Additional file 1:
**Table S1.** Baseline and intra-operative characteristics of patients with ejection fraction ≤30 % who underwent cardiac surgery: comparisons between survived and dead patients. **Table S2.** Post-operative complications and outcome data of patients with ejection ≤30 % who underwent cardiac surgery: comparisons between survived and dead patients. **Table S3.** Baseline comorbidities and information of patients undergoing mitral valve surgery. (DOCX 32 kb)

## Abbreviations

ACEF: Age-creatinine-ejection fraction
AF: Atrial fibrillation
AKI: Acute kidney injury
CABG: Coronary artery bypass graft
COPD: Chronic obstructive pulmonary disease
EF: Ejection fraction
eGFR: Estimated glomerular filtration rate
IABP: Intra-aortic balloon pump
ICU: Intensive care unit
LCOS: Low cardiac output syndrome
LOS: Length of stay
LV: Left ventricle
LVEF: Left ventricular ejection fraction
RRT: Renal replacement therapy
