# Supplementary material for: Outcome of cardiac surgery in patients with low preoperative ejection fraction
Source: BMC Anesthesiol. 2016 Oct 18;16:97. doi: 10.1186/s12871-016-0271-5 (PMC5069974; doi:10.1186/s12871-016-0271-5)
Supplement: Additional file 1: — Table S1. Baseline and intra-operative characteristics of patients with ejection fraction ≤30 % who underwent cardiac surgery: comparisons between survived and dead patients. Table S2. Post-operative complications and outcome data of patients with ejection ≤30 % who underwent cardiac surgery: comparisons between survived and dead patients. Table S3. Baseline comorbidities and information of patients undergoing mitral valve surgery. (DOCX 32 kb) [file 12871_2016_271_MOESM1_ESM.docx]

**aDDITIONAL FILE 1**

**Outcome of Cardiac Surgery in Patients with Low Preoperative Ejection Fraction**

M. Pieri, A. Belletti, F. Monaco, A. Pisano, M. Musu, V. Dalessandro,

G. Monti, G. Finco, A. Zangrillo, G. Landoni

**Table S1** page 2

**Table S2** page 5

**Table S3** page 7

**Table S1.** Baseline and intra-operative characteristics of patients with ejection fraction ≤30% who underwent cardiac surgery: comparisons between survived and dead patients.

| **Variable** | **Total (N = 290)** | **Survivors (N = 268)** | **Dead (N = 22)** | **p-value** |
| --- | --- | --- | --- | --- |
| **Preoperative characteristics** | | | | |
| Gender (Male), n | 239 (82%) | 227 (85%) | 12 (55%) | < 0.001 |
| Age, years | 65.4 ± 9.9 | 65.1 ± 9.8 | 69.5 ± 10.1 | 0.04 |
| Height, cm | 170.0 ± 8.1 | 170.2 ± 7.9 | 166.9 ± 10.3 | 0.2 |
| Weight, kg | 72.3 ± 12.9 | 72.5 ± 12.9 | 69.2 ± 12.5 | 0.7 |
| BMI | 25.0 ± 3.8 | 25.0 ± 3.8 | 24.8 ± 4.1 | 0.9 |
| Comorbidity |  |  |  |  |
| > COPD, n | 102 (35%) | 89 (33%) | 13 (59%) | 0.01 |
| > Preoperative EF, % | 26.9 ± 3.8 | 26.9 ± 3.9 | 26.7 ± 3.5 | 0.6 |
| > Peripheral vasculopathy, n | 83 (29%) | 77 (29%) | 6 (27%) | 0.9 |
| > Arterial hypertension, n | 147 (51%) | 138 (51%) | 9 (41%) | 0.3 |
| > Type II diabetes mellitus, n | 64 (22%) | 63 (24%) | 1 (4.5%) | 0.057 |
| > Carotid stenosis, n | 26 (9%) | 25 (9.3%) | 1 (4.5%) | 0.7 |
| > Angina, n | 43 (15%) | 38 (14%) | 5 (23%) | 0.3 |
| > Previous AMI, n | 105 (36%) | 99 (37%) | 6 (27%) | 0.4 |
| > Previous TIA or stroke, n | 27 (9.3%) | 26 (9.7%) | 1 (4.5%) | 0.7 |
| > Previous vascular surgery, n | 12 (4.1%) | 12 (4.5%) | 0 (0%) | 0.6 |
| > Standard EuroSCORE | 7 (5 - 9) | 7 (5 – 9) | 9 (6 – 10) | 0.04 |
| > ACEF score | 2.6 (2.32 - 3.04) | 2.57 (2.3 – 3) | 3.16 (2.53 – 3.44) | 0.025 |
| > ACEF risk | 10.19 (7.42 - 16.33) | 9.82 (7.26 – 15.71) | 18.52 (9.46 – 24.34) | 0.025 |
| > Endocarditis, n | 10 (3.4%) | 9 (3.4%) | 1 (4.5%) | 0.6 |
| > Creatinine clearance, ml/h | 63.34 (47.68 - 78.05) | 63.6 (48.9 – 78.2) | 48 (39.7 – 66.5) | 0.12 |
| > Renal failure, n | 63 (22%) | 54 (20%) | 9 (41%) | 0.02 |
| > Dialysis, n | 4 (1.4%) | 4 (1.5%) | 0 (0%) | 0.99 |
| NYHA |  |  |  | 0.004 |
| > I | 16 (5.5%) | 16 (6%) | 0 (0%) |  |
| > II | 65 (22%) | 63 (24%) | 2 (9.1%) |  |
| > III | 115 (40%) | 110 (41%) | 5 (23%) |  |
| > IV | 20 (6.9%) | 15 (5.6%) | 5 (23%) |  |
| Timing of surgery |  |  |  | 0.7 |
| > Emergency, n | 10 (3.4%) | 9 (3.4%) | 1 (4.5%) |  |
| > Urgency, n | 59 (20%) | 54 (20%) | 5 (23%) |  |
| > Election, n | 221 (76%) | 205 (76%) | 16 (73%) |  |
| Redo surgery, n | 24 (8.3%) | 22 (8.2%) | 2 (9.1%) | 0.7 |
| Preoperative IABP, n | 86 (30%) | 76 (28%) | 10 (45%) | 0.09 |
| Preoperative inotropes, n | 8 (2.8%) | 6 (2.2%) | 2 (9.1%) | 0.12 |
| Chronic therapy |  |  |  |  |
| > Antiplatelets, n | 99 (34%) | 94 (35%) | 5 (23%) | 0.3 |
| > Diuretics, n | 210 (72%) | 191 (71%) | 19 (86%) | 0.15 |
| > Beta-blockers, n | 136 (47%) | 130 (49%) | 6 (27%) | 0.055 |
| > Antibiotics, n | 16 (5.5%) | 14 (5.2%) | 2 (9.1%) | 0.3 |
| > Calcium channel blockers, n | 44 (15%) | 41 (15%) | 3 (14%) | 0.99 |
| > Nitrates, n | 88 (30%) | 81 (30%) | 7 (32%) | 0.9 |
| > ACE inhibitors, n | 201 (69%) | 188 (70%) | 13 (59%) | 0.3 |
| > Oral anticoagulants, n | 46 (16%) | 43 (16%) | 3 (14%) | 0.99 |
| > Heparin, n | 23 (7.9%) | 20 (7.5%) | 3 (14%) | 0.4 |
| Creatinine, mg/dl | 1.3 ± 1.1 | 1.3 ± 1.1 | 1.3 ± 0.5 | 0.2 |
| Bilirubin, mg/dl | 0.82 (0.6 - 1.03) | 0.81 (0.6 – 1) | 0.96 (0.8 – 2.8) | 0.1 |
| **Surgical interventions** | | | | |
| CABG, n | 140 (29%) | 133 (30%) | 7 (18%) | 0.11 |
| > Isolated CABG, n | 59 (12%) | 59 (13%) | 0 (0%) | 0.01 |
| Mitral valve surgery, n | 105 (22%) | 91 (21%) | 14 (37%) | 0.005 |
| > Isolated mitral valve surgery, n | 33 (6.9%) | 28 (6.4%) | 5 (13%) | 0.09 |
| > Mitral valve replacement, n | 41 (8.6%) | 35 (8%) | 6 (16%) | 0.066 |
| > Mitral valve repair, n | 64 (13%) | 56 (13%) | 8 (21%) | 0.09 |
| Aortic valve surgery, n | 89 (19%) | 82 (19%) | 7 (18%) | 0.9 |
| > Isolated aortic valve surgery, n | 27 (5.7%) | 25 (5.7%) | 2 (5.3%) | 0.99 |
| > Aortic valve replacement, n | 89 (19%) | 82 (19%) | 7 (18%) | 0.9 |
| > Aortic valve repair, n | 0 (0%) | 0 (0%) | 0 (0%) |  |
| Tricuspid valve surgery, n | 28 (5.9%) | 26 (5.9%) | 2 (5.3%) | 0.99 |
| > Isolated tricuspid valve surgery, n | 1 (0.21%) | 1 (0.23%) | 0 (0%) | 0.99 |
| > Tricuspid valve replacement, n | 2 (0.42%) | 2 (0.46%) | 0 (0%) | 0.99 |
| > Tricuspid valve repair, n | 26 (5.5%) | 24 (5.5%) | 2 (5.3%) | 0.99 |
| Pulmonic valve surgery, n | 1 (0.21%) | 1 (0.23%) | 0 (0%) | 0.99 |
| > Isolated pulmonic valve surgery, n | 1 (0.21%) | 1 (0.23%) | 0 (0%) | 0.99 |
| Surgery on ascending aorta, n | 31 (6.5%) | 27 (6.2%) | 4 (11%) | 0.3 |
| > Isolated surgery on ascending aorta, n | 3 (0.63%) | 3 (0.68%) | 0 (0%) | 0.99 |
| Left ventricle surgery, n | 36 (7.6%) | 35 (8%) | 1 (2.6%) | 0.3 |
| > Isolated left ventricle surgery, n | 6 (1.3%) | 6 (1.4%) | 0 (0%) | 0.99 |
| **Intraoperative management** | | | | |
| CPB, n | 255 (90%) | 235 (88%) | 20 (91%) | 0.2 |
| Duration of aortic cross clamping, min | 60 (47 - 77) | 60 (46.5 – 76.5) | 67 (53 – 77.5) | 0.3 |
| Duration of CPB, min | 86 (67 - 105) | 86 (65 – 105) | 101 (90 – 114) | 0.14 |

ACEF: age-creatinine-ejection fraction; AMI: acute myocardial infarction; BMI: body mass index; CABG: coronary artery bypass graft; COPD: chronic obstructive pulmonary disease; CPB: cardiopulmonary bypass; EF: ejection fraction; IABP: intra-aortic balloon pump; NYHA: New York Heart Association; TIA: transient ischemic attack

**Table S2.** Post-operative complications and outcome data of patients with ejection ≤30% who underwent cardiac surgery: comparisons between survived and dead patients.

| **Variable** | **Total (N = 290)** | **Survived (N = 268)** | **Dead (N = 22)** | **p-value** |
| --- | --- | --- | --- | --- |
| **Post-operative complications** | | | | |
| Post-operative AMI, n | 4 (1.4%) | 4 (1.5%) | 0 (0%) | 0.99 |
| Post-operative peak troponin value, ng/ml | 8.78 (4.25 - 15.03) | 8.03 (4.15 – 14.4) | 14.58 (8.87 – 24.3) | 0.01 |
| Post-operative AF, n | 84 (29%) | 78 (29%) | 6 (27%) | 0.9 |
| LCOS, n | 123 (42%) | 106 (40%) | 17 (77%) | 0.001 |
| Inotropes more than 48 hours, n | 108 (37%) | 94 (35%) | 14 (64%) | 0.008 |
| Cardiogenic shock, n | 22 (7.6%) | 7 (2.6%) | 15 (68%) | < 0.001 |
| Post-operative peak creatinine value, mg/dl | 1.3 ± 0.9 | 1.3 ± 0.9 | 1.3 ± 0.5 | 0.4 |
| AKI, n | 89 (31%) | 70 (26%) | 19 (86%) | < 0.001 |
| RRT, n | 17 (5.9%) | 8 (3%) | 9 (41%) | < 0.001 |
| Bleeding in the first 12 postoperative hours, ml | 270 (200 - 380) | 270 (200 – 380) | 270 (200 – 425) | 0.7 |
| Total post-operative bleeding, ml | 500 (350 - 800) | 500 (350 – 800) | 580 (410 – 750) | 0.8 |
| Need for blood products transfusion, n | 113 (39%) | 97 (36%) | 16 (73%) | < 0.001 |
| RBC transfusions, n of units per patient | 0 (0 - 2) | 0 (0 – 1) | 3.5 (0 – 6) | < 0.001 |
| FFP transfusions, n of units per patient | 0 (0 - 0) | 0 (0 – 0) | 0 (0 – 3) | < 0.001 |
| PLT transfusions, n of units per patient | 0 (0 - 0) | 0 (0 – 0) | 0 (0 – 4) | < 0.001 |
| Neurological damage type 1, n | 5 (1.7%) | 5 (1.9%) | 0 (0%) | 0.99 |
| Neurological damage type 2, n | 7 (2.4%) | 7 (2.6%) | 0 (0%) | 0.99 |
| Severe pulmonary dysfunction, n | 16 (5.5%) | 11 (4.1%) | 5 (23%) | 0.004 |
| Tracheostomy, n | 13 (4.5%) | 4 (1.5%) | 9 (41%) | < 0.001 |
| Need for re-intubation, n | 10 (3.4%) | 6 (2.2%) | 4 (18%) | 0.004 |
| Sepsis, n | 12 (4.1%) | 10 (3.7%) | 2 (9.1%) | 0.2 |
| Mediastinitis, n | 3 (1.03%) | 2 (0.75%) | 1 (4.5%) | 0.2 |
| **Outcome data** | | | | |
| Duration of MV, hours | 25 (15 - 71) | 24 (15 – 60) | 72 (16 – 120) | 0.08 |
| ICU stay, days | 4 (2 - 6) | 4 (2 – 6) | 9 (3 – 19) | 0.01 |
| Hospital stay, days | 8 (6 - 12) | 7 (6 – 11) | 14 (4.5 – 26) | 0.2 |
| Death, n | 22 (7.6%) |  |  |  |

AF: atrial fibrillation; AKI: acute kidney injury; AMI: acute myocardial infarction; FFP: fresh frozen plasma; ICU: intensive care unit; LCOS: low cardiac output syndrome; MV: mechanical ventilation; PLT: platelets; RBC: red blood cells; RRT: renal replacement therapy

**Table S3.** Baseline comorbidities and information of patients undergoing mitral valve surgery

| **Parameter** | **EF ≤ 40%  (n = 282)** | **EF ≤ 30%  (n = 105)** |
| --- | --- | --- |
| COPD, n | 87 (31%) | 39 (37%) |
| Cirrhosis, n | 4 (1.4%) | 2 (1,9%) |
| Type 2 Diabetes mellitus, n | 41 (14%) | 20 (19%) |
| ACEF score | 2.14 ± 0.57 | 2.61 ± 0.49 |
| ACEF risk | 7.4 ± 6 | 11.7 ± 7.1 |
| Creatinine clearance, ml/h | 67 ± 26 | 63 ± 23 |
| Peripheral vasculopathy, n | 60 (21%) | 25 (24%) |
| Arterial hypertension, n | 147 (52%) | 56 (53%) |
| Carotid stenosis, n | 19 (7%) | 7 (6,7%) |
| Angina, n | 29 (10%) | 13 (12%) |
| Previous AMI, n | 66 (23%) | 31 (30%) |
| Previous TIA or stroke, n | 124 (44%) | 51 (49%) |
| Previous vascular surgery, n | 10 (3.5%) | 4 (3.8%) |
| Standard EuroSCORE | 6,61 ± 3,05 | 7.46 ± 3.49 |
| Endocarditis, n | 10 (3.6%) | 6 (5.7%) |
| Preoperative IABP, n | 76 (27%) | 48 (45.7%) |
| Redo surgery, n | 42 (15%) | 10 (9.5%) |

ACEF: age-creatinine-ejection fraction; AMI: acute myocardial infarction; COPD: chronic obstructive pulmonary disease; IABP: intra-aortic balloon pump; TIA: transient ischemic attack
